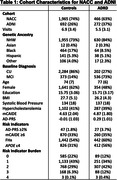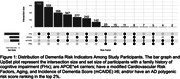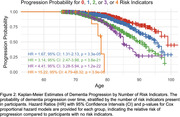# Evaluating the Impact of Combined Clinical and Genetic Risk Factors on Dementia Onset

**DOI:** 10.1002/alz.086576

**Published:** 2025-01-03

**Authors:** Shea J Andrews, Caroline Warly Solsberg, Alan E. Renton, Brian Fulton‐Howard, Jennifer S. Yokoyama, Kristine Yaffe

**Affiliations:** ^1^ University of California, San Francisco, San Francisco, CA USA; ^2^ University of California San Francisco, San Francisco, CA USA; ^3^ Icahn School of Medicine at Mount Sinai, New York, NY USA; ^4^ Departments of Psychiatry and Behavioral Sciences, Neurology, and Epidemiology, University of California San Francisco, San Francisco, CA USA

## Abstract

**Background:**

Integrating clinical and genetic risk factors for dementia in a precision medicine framework can play a crucial role in primary prevention. Here, we ascertained the proportion of individuals who are at heightened risk of developing dementia based on their family history, genetic, and clinical risk factors and evaluated how the additive burden of these risk indicators is associated with incident dementia.

**Method:**

We analyzed longitudinal data from 3,395 diverse older adults, dementia‐free at baseline with follow‐up and whole genome sequencing, enrolled in the National Alzheimer’s Co‐coordinating Center and the Alzheimer’s Disease Neuroimaging Initiative (Table 1). Our risk indicators comprised: 1) self‐reported first‐degree relative with cognitive impairment; 2) carriage of the APOE*e4 allele; 3) an AD polygenic risk score, incorporating 24 variants and normalized for genetic ancestry, ranking in the top 2% (AD‐PRS); 4) presence of dominantly inherited AD mutations in *PSEN1*, *PSEN2*, or *APP*; and 5) a score of ≥6 on a modified Cardiovascular Risk Factors, Aging, and Incidence of Dementia Score (mCAIDE), comprised of age, sex, education, obesity, hypertension, and hypercholesterolemia. We calculated the total risk burden as the sum of these indicators. Cox proportional hazard models were employed to estimate the risk of dementia progression associated with increasing risk burden.

**Results:**

Among participants, 55% had a family history of dementia, 36% were APOE*e4 carriers, 33% had high mCAIDE scores, 2.2% had extreme AD‐PRS, and none had ADAD mutations. Distribution of risk indicators was as follows: 20% had none, 41% one, 32% two, 7.5% three, and 0.2% four (Figure 1). Our analysis revealed a graded increase in dementia risk with more indicators: compared to those with none, individuals with one, two, three, or four indicators had 1.67, 3.14, 4.41, and 15.22 times increased dementia risk, respectively (Figure 2).

**Conclusion:**

The majority of our cohort exhibited at least one dementia risk indicator, with a higher risk burden markedly increasing dementia risk. These findings underscore the value of integrating genetic and clinical risk factors in predicting dementia, highlighting the potential of precision medicine in guiding preventive strategies for at‐risk individuals.